# Supplementary material for: A Critical View of the Application of the APEX Software (Aqueous Photochemistry of Environmentally-Occurring Xenobiotics) to Predict Photoreaction Kinetics in Surface Freshwaters
Source: Molecules. 2019 Dec 18;25(1):9. doi: 10.3390/molecules25010009 (PMC7017383; doi:10.3390/molecules25010009)
Supplement: Supplementary file 1 [file molecules-25-00009-s001.zip › 3.2.4_gcc-4.4.0.zip!/3.2.4_gcc-4.4.0/3.2.4_gcc-4.4.0/doc/octave/html/interpreter/The-_003ccode_003edo_002duntil_003c_002fcode_003e-Statement.html]

The @code{do-until} Statement - Untitled


Next: The <code>for</code> Statement,
Previous: The <code>while</code> Statement,
Up: Statements


---

### 10.4 The `do-until` Statement

The `do-until` statement is similar to the `while` statement,
except that it repeatedly executes a statement until a condition becomes
true, and the test of the condition is at the end of the loop, so the
body of the loop is always executed at least once. As with the
condition in an `if` statement, the condition in a `do-until`
statement is considered true if its value is non-zero, and false if its
value is zero. If the value of the conditional expression in a
`do-until` statement is a vector or a matrix, it is considered
true only if it is non-empty and *all* of the elements are non-zero.

Octave's `do-until` statement looks like this:

```
     do
       body
     until (condition)
```

Here body is a statement or list of statements that we call the
body of the loop, and condition is an expression that
controls how long the loop keeps running.

This example creates a variable `fib` that contains the first ten
elements of the Fibonacci sequence.

```
     fib = ones (1, 10);
     i = 2;
     do
       i++;
       fib (i) = fib (i-1) + fib (i-2);
     until (i == 10)
```

A newline is not required between the `do` keyword and the
body; but using one makes the program clearer unless the body is very
simple.
